# Deferral of non-emergency cardiac procedures is associated with increased early emergency cardiovascular hospitalizations

**DOI:** 10.1007/s00392-022-02032-z

**Published:** 2022-05-23

**Authors:** Stefanie Andreß, Tilman Stephan, Dominik Felbel, Alex Mack, Michael Baumhardt, Johannes Kersten, Dominik Buckert, Alexander Pott, Tillman Dahme, Wolfgang Rottbauer, Armin Imhof, Manuel Rattka

**Affiliations:** Klinik für Innere Medizin II, Universitätsklinikum Ulm, Albert Einstein Allee 23, 89081 Ulm, Germany

**Keywords:** COVID-19, Epidemiology, Deferral, Outcome, Hospitalization, Valvular heart disease

## Abstract

**Background:**

During the COVID-19 pandemic, in anticipation of a demand surge for high-care hospital beds, many hospitals postponed non-emergency interventions of cardiac patients.

**Aim:**

The aim of this study was to assess the outcomes of cardiac patients whose non-emergency interventions had been deferred during the COVID-19 pandemic.

**Methods:**

Patients whose non-emergency cardiac intervention had been cancelled between March 19th and April 30th, 2020 were included (study group). All patients were considered as deferrable according to current recommendations. Patients’ outcomes after 12 months were compared to a seasonal control group who underwent non-emergency interventions in 2019 as scheduled. The primary endpoint was a composite of emergency cardiovascular hospitalization and death. Secondary endpoints were levels of symptoms and cardiac biomarkers.

**Results:**

Outcomes of 193 consecutive patients in the study group were assessed and compared to 216 controls. The primary endpoint occurred significantly more often in the study group (HR 2.42, 95%CI 1.63–3.61, *p* < 0.001). This was driven by an increase in hospitalizations. Subgroup analyses showed that especially patients with a deferred transcatheter heart valve intervention experienced early emergency hospitalization (HR 9.55, 95%CI 3.70–24.62, *p* < 0.001). These findings were accompanied by more pronounced symptoms and higher biomarker levels.

**Conclusions:**

Deferral of non-emergency cardiac interventions to meet the higher demand for hospital beds during the COVID-19 crisis is associated with early emergency cardiovascular hospitalizations. Patients suffering from valvular heart disease especially constitute a vulnerable group. Consequently, our results suggest that current recommendations on the management of cardiovascular disease during the COVID-19 pandemic need revision.

**Graphical abstract:**

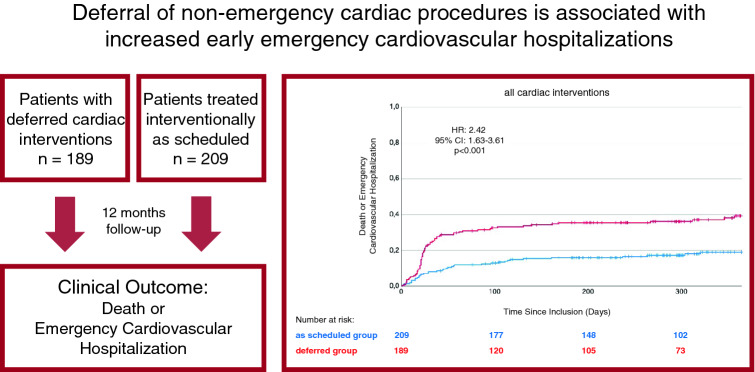

**Supplementary Information:**

The online version contains supplementary material available at 10.1007/s00392-022-02032-z.

## Introduction

Since the beginning of the COVID-19 pandemic and due to the wave-like undulating incidence rates, healthcare systems worldwide have exceeded their treatment capacities for those infected with SARS-CoV-2 several times. Especially, beds at intermediate care (IMC) and intensive care units (ICU) were and still are desperately needed in many regions. In spring 2020, in anticipation of this surge in demand, healthcare professionals recommended reducing the number of non-emergency hospital admissions and postponing low-priority medical interventions. This affected many medical specialties, interventional cardiology amongst others. Therefore, cardiac societies from around the world developed recommendations to distinguish between urgent and postponable cardiac interventions, resulting in the deferral of appointments [1]. However, the effect of this approach on patients’ outcomes has not yet been evaluated.

## Methods

### Study design

We conducted an observational case–control study considering cardiac patients treated at the Department of Medicine II at Ulm University Medical Center, Ulm, Germany, whose non-emergency appointments had been cancelled, postponed, or deferred during the initial phase of the COVID-19 pandemic. Patients eligible for inclusion were those receiving a non-emergency intervention due to (1) severe valve stenosis or insufficiency, (2) (suspected) significant coronary artery disease (CAD), (3) atrial or ventricular arrhythmia, and (4) patients who received an implantable cardioverter defibrillator (ICD) or a permanent pacemaker. Patients with a scheduled non-emergency intervention between March 19th, 2020, and April 30th, 2020, which had been deferred, were selected as the study group. All patients in the study group were in a condition allowing the postponement of procedures and were categorized either as “lower priority” or “elective” according to ESC recommendations [1]. Non-emergency patients admitted between March 19th, 2019, and April 30th, 2019, served as the seasonal control group. The study complies with the Declaration of Helsinki and was approved by the local ethics committee. This study adheres to the STROBE statement.

### Data collection, follow-up, and laboratory procedures

Demographic, clinical and laboratory data, at baseline, at the day of intervention and follow-up, and outcome data were extracted from our patient management system by two physicians (SA und MR) and adjudicated by a third one (TS) in case of any kind of difference. Patients were scheduled for outpatient clinic visits including clinical assessment and 12-lead ECG at 1, 3, and 6 months after the procedure, and thereafter every 6 months, as part of our clinical routine, whenever possible. Left-ventricular ejection fraction (LVEF) was measured either by echocardiography (EPIQ 7, Koninklijke Philips N.V., Eindhoven, The Netherlands) or cardiac ventriculography during cardiac catheterization and graded as normal (0), mildly impaired (1), moderately impaired (2), or severely impaired (3), according to guideline specific recommendations [2]. Blood samples were drawn at the time of hospital admission or at the outpatient clinic visits for measurements of high-sensitivity cardiac troponin T (hs cTnT), NT-pro BNP, and creatinine (ElectroChemiLumineszenz ImmunoAssay “ECLIA” Roche, Cobas 8000, Modul e801 and e601) as part of the clinical routine.

### Primary and secondary endpoints

The primary endpoint was a composite of emergency cardiovascular hospitalization or death. Secondary endpoints were heart failure symptoms as measured by NYHA class, level of angina pectoris as measured by CCS class, plasma NT-pro BNP levels, plasma hs cTnT levels, and LVEF. The start of the follow-up period was defined as the day of the scheduled intervention.

### Statistical analysis

Continuous variables were described as mean ± standard deviation or median together with interquartile range (IQR), as appropriate. Normal distribution was assessed by the Kolmogorov–Smirnov test. If a metric variable was not normally distributed at baseline or at the end of follow-up, both were presented as median together with the IQR for better comparability. Ordinal variables were described as median together with interquartile range (IQR). For some variables (NYHA class, CCS class, LVEF), mean ± standard deviation was displayed for reasons of clarity and comprehensibility. Categorical variables were presented as number (percent). Student’s *t* tests, the Mann–Whitney *U*-Test, or the *χ*^2^ test was used to compare variables between the study group and control group, where appropriate. The Kaplan–Meier estimator was used to assess the time to event and groups were compared using the Cox proportional hazard model. Parameters with a* p* value < 0.05 were considered to be statistically significant. Statistical assessment was performed by SPSS Statistics 25 software (Version 2017, IBM, Armonk, NY, USA). Due to the explorative nature of this study, all results from statistical tests must be interpreted as hypothesis generating.

## Results

### Study population

Between March 19th, 2020, and April 30th, 2020, cardiac interventions of 193 patients were cancelled, postponed, or deferred at our tertiary care center. 78 patients (40.4%; study group) had been scheduled for cardiac catheterization, 50 patients (25.9%) for transcatheter heart valve interventions and 65 patients (33.7%) for rhythmological cardiac interventions. In the reference period between March 19th, 2019, and April 30th, 2019, a total of 216 patients (control group) underwent cardiac interventions as scheduled. 94 patients (43.5%) underwent cardiac catheterization, 48 patients (22.2%) had a heart valve intervention, and 74 patients (34.3%) a rhythmological cardiac intervention. Patient outcomes were assessed for the subsequent 12 months following the original date of the scheduled non-emergency cardiac intervention. A total of 11 patients were lost to follow-up (study group: 4 patients; control group: 7 patients). Consequently, the characteristics of 189 patients from the study group and 209 patients from the control group were compared (Table 1, Supplementary Table 1). At the baseline, patients’ mean age was slightly above 70 years, and most patients in both groups were of the male sex. We observed a significantly higher median heart rate at the baseline in patients of the study group (73 [63, 85] beats per minute (bpm)) compared to the control group (68 [60, 80] bpm; *p* = 0.010). Additionally, significantly more patients in the control group had a positive family history for cardiovascular disease (control group: 55 out of 209 patients; study group: 23 out of 154 patients; *p* = 0.009). Otherwise, there were no significant differences in the baseline patient characteristics (Table 1).

**Table 1:** Patient characteristics at baseline

	Control group	Study group	*p* Value
	*n* = 209	*n* = 189	
Age (years)	71 ± 13	72 ± 11	0.271
Male sex	139 (67)	113 (60)	0.165
Height (cm)	171 ± 9	173 ± 10	0.357
Weight (kg)	84 ± 18	86 ± 19	0.428
Heart rate (bpm)	68 [60, 80]	73 [63, 85]	**0.010**
Blood pressure systolic (mmHg)	130 ± 22	130 ± 19	0.931
Blood pressure diastolic (mmHg)	79 ± 13	76 ± 14	0.502
Arterial hypertension	165 (79)	121 (78)**	0.839
Dyslipidemia	148 (71)	97 (63)*	0.116
Diabetes mellitus	48 (23)	46 (30)*	0.138
Family history for CVD	55 (26)	23 (15)*	**0.009**
Smoker	75 (36)	54 (35)*	0.872
Obesity	52 (25)	34 (22)*	0.535
History of TIA/stroke	18 (9)	17 (11)*	0.439
COPD	11 (5)	13 (8)*	0.228
OSAS	11 (5)	11 (7)**	0.468
CKD	36 (17)	29 (19)**	0.715
Known CAD	138 (66)	116 (75)**	0.070
Known arrhythmia	119 (57)	97 (63)**	0.278

Bold denotes significant *p*- values

Values are shown as mean ± SD, median [IQR], or as number (%)

*bpm* beats per minute, *CVD* cardiovascular disease, *COPD* chronic pulmonary obstructive disease, *OSAS* obstructive sleep apnea syndrome, *CKD* chronic kidney disease, *CAD* coronary artery disease

*Characteristics of 154 patients were assessed; **characteristics of 155 patients were assessed

### Outcome: cardiovascular hospitalization and mortality

**Fig. 1:**
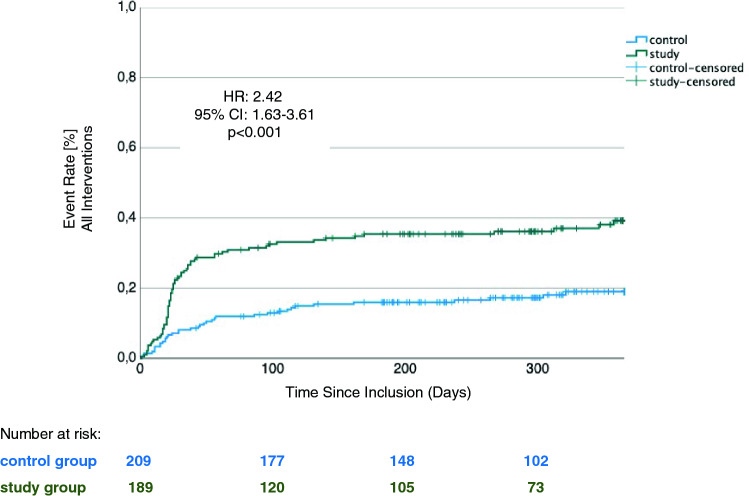
Kaplan–Meier estimator of the time to emergency cardiovascular hospitalization or death (all interventions)

A primary endpoint event occurred in 70 patients (37.0%) in the study group and in 37 patients (17.7%) in the control group (HR for study vs control group: 2.42, 95% CI 1.63–3.61, *p* < 0.001; Fig. 1). 30-Day Kaplan–Meier event rates were 13.4% for the study group and 8.1% for the control group. 60-Day Kaplan–Meier event rates were 29.2% for the study group and 12.0% for the control group. In the study group there were eight fatalities, whereas in the control group, there were six. All deaths occurred after a hospitalization event; thus, death did not contribute to the combined primary endpoint.

### Outcome: clinical characteristics

To evaluate the effect of the deferral of non-emergency cardiac interventions on the patients’ clinical course, we assessed their level of angina pectoris, as measured by CCS class, and dyspnoea, as measured by NYHA class, at baseline and after 12 months of follow-up. Initially, there were no differences in symptoms between both groups (Table 2, Supplementary Fig. 1A and B). By contrast, at the end of the follow-up period, there was a significant difference in the CCS class (study group: 0.5 ± 1.1, control group: 0.3 ± 0.8; *p* = 0.011; Table 2, Supplementary Fig. 1B). Whereas symptoms of angina pectoris ameliorated significantly in the control group (baseline: 0.8 ± 1.2, follow-up: 0.3 ± 0.8, *p* < 0.001), there was no difference in the study group (Table 2; Supplementary Fig. 1B). As for the NYHA class, the course of symptoms of dyspnoea only improved significantly in the control group (baseline: 1.9 ± 0.8, follow-up: 1.5 ± 0.7, *p* < 0.001; Table 2, Supplementary Fig. 1A), too.

**Table 2:** Clinical characteristics at baseline and follow-up

	Control group	Study group	*p* Value
	*n* = 209	*n* = 189	
NYHA class	***		
Baseline	1.9 ± 0.8	1.9 ± 0.8	0.412
Follow-up	1.5 ± 0.7	1.7 ± 0.9	0.084
CCS class	***		
Baseline	0.8 ± 1.2	0.7 ± 1.1	0.281
Follow-up	0.3 ± 0.8	0.5 ± 1.1	**0.011**
NT-pro BNP (pg/ml)			
Baseline	588 [177, 1611]	877 [227, 2115]	0.252
Follow-up	483 [211, 1149]	832 [218, 3199]	**0.021**
Troponin T (ng/L)			
Baseline	16 [9, 28]	20 [10, 35]	0.094
Follow-up	16 [9, 25]	23 [11, 42]	**0.010**
LVEF	***	*	
Baseline	2.1 ± 1.2	2.3 ± 1.2	0.167
Follow-up	1.8 ± 1.1	2.1 ± 1.2	**0.036**
Creatinine (µmol/L)			
Baseline	108 ± 94	103 ± 52	0.694
Follow-up	120 ± 125	110 ± 52	0.418

Bold denotes significant *p*- values

Values are shown as mean ± SD or median [IQR]

*NYHA* New York Heart Association, *CCS* Canadian Cardiovascular Society, *LVEF* left-ventricular ejection fraction

*p < .05 versus baseline; **p < .01 versus baseline; ***p < .001 versus baseline

Evaluation of serum cardiac biomarker levels showed that median NT-pro BNP (control group: 483 [211, 1149] pg/ml, study group: 832 [218, 3199] pg/ml; *p* = 0.021), and troponin T levels (control group: 16 [9, 25] ng/L, study group: 23 [11, 42] ng/L; *p* = 0.010) were significantly lower in the control group than in the study group at the 12-months follow-up (Table 2). We could not detect differences between the baseline and follow-up biomarker levels for each group, respectively.

Furthermore, assessment of left-ventricular function demonstrated a significantly better left-ventricular systolic function in the control group at the end of the follow-up phase (control group: 1.8 ± 1.1, study group: 2.1 ± 1.2; *p* = 0.036). This was related to an improvement in contractile function in the study group (baseline: 2.1 ± 1.2, follow-up: 1.8 ± 1.1; *p* < 0.001; Table 2, Supplementary Fig. 2).

### Subgroup analyses

Since our analyses so far had been comprised of the outcomes of patients suffering from different types of cardiac disease, we next examined the subgroups in more detail.

**Fig. 2:**
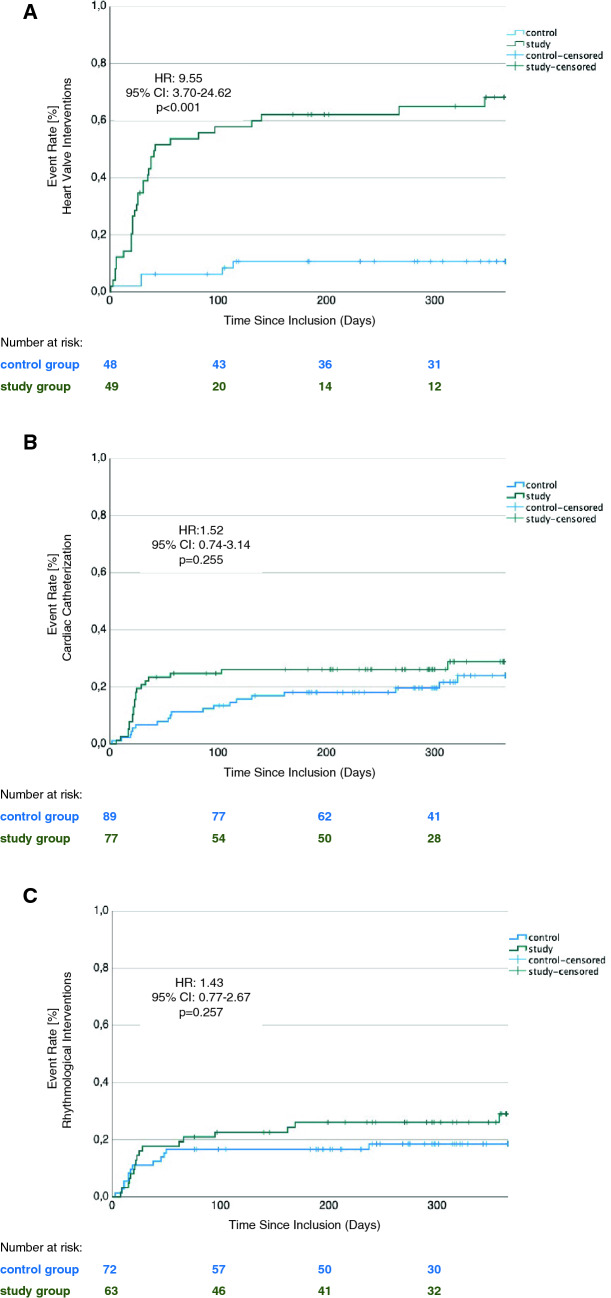
Kaplan–Meier estimators of the time to emergency cardiovascular hospitalization or death of patients undergoing **A** a transcatheter heart valve intervention, **B** cardiac catheterization, and **C** a rhythmological intervention

A total of 97 patients had severe heart valve stenosis or insufficiency (control group: 48 patients, study group: 49 patients) and were scheduled for transcatheter heart valve replacement or repair (Supplementary Tables 1 and 2). A primary endpoint event occurred in 32 patients (65.3%) in the study group and in 5 patients (10.4%) in the control group (HR 9.55, 95% CI 3.70–24.62, *p* < 0.001; Fig. 2A). This was driven by both patients initially scheduled for transcatheter aortic valve implantation (TAVI), and patients whose non-emergency percutaneous mitral valve repair (PMVR) intervention had been deferred (Supplementary Fig. 3A and B).

In patients scheduled for transcatheter heart valve replacement or repair, 30-day Kaplan–Meier event rates were 34.7% for the study group and 6.2% for the control group. 60-Day Kaplan–Meier event rates were 53.7% for the study group and 6.2% for the control group. This was associated with a significant increase in median NT-pro BNP levels in the study group (baseline: 1447 [701, 2293] pg/ml, follow-up 3307 [2079, 5276] pg/ml; *p* = 0.038). The NYHA class after 12 months of follow-up did not show any improvement. In contrast, patients in the control group showed ameliorated symptoms as measured by NYHA class (baseline: 2.4 ± 0.7, follow-up: 1.5 ± 0.8; *p* < 0.001) and CCS class (baseline: 0.9 ± 1.0, follow-up 0.2 ± 0.7; *p* = 0.001). Moreover, in the control group, left-ventricular systolic function significantly improved (baseline: 2.0 ± 1.3, follow-up: 1.8 ± 1.1; *p* = 0.045), too. Clinical characteristics are shown in Supplementary Table 3 in more detail.

As for patients scheduled for cardiac catheterizations (166 patients; Supplementary Tables 1 and 4) or electrophysiological cardiac interventions (135 patients; Supplementary Tables 1 and 6), we could not demonstrate significant differences regarding the primary endpoint (Fig. 2B and C). However, patients who had undergone coronary catheterization as scheduled showed significantly ameliorated symptoms of dyspnoea (NYHA class baseline: 1.8 ± 0.8, follow-up: 1.5 ± 0.7; *p* = 0.006) and angina pectoris (CCS class baseline: 1.1 ± 1.3, follow-up: 0.4 ± 1.0; *p* = 0.006). Additionally, we detected significantly lower levels of troponin T (baseline: 15 [9, 27], follow-up: 14 [7, 23]; *p* = 0.017) and an improvement in left-ventricular systolic function (baseline: 2.1 ± 1.1, follow-up: 1.8 ± 1.0; *p* < 0.001) in the control group (Supplementary Table 5). Patients whose electrophysiological intervention had been postponed showed an increase in symptoms of dyspnoea (baseline: 1.4 ± 0.6, follow-up: 1.5 ± 0.7; *p* = 0.033), while the NYHA class of the control group ameliorated significantly (baseline: 1.8 ± 0.8, follow-up: 1.4 ± 0.7; *p* = 0.012; Supplementary Table 7).

### Comparison of clinical characteristics at baseline and at the time of the actual transcatheter heart valve intervention of deferred patients

Our results suggest that the deferral of transcatheter heart valve interventions is in particular associated with the progression of disease, which, consequently, results in early emergency cardiovascular hospitalization. To substantiate this hypothesis, we assessed and compared clinical characteristics at the baseline and at the time of the actual intervention of patients whose heart valve intervention was initially cancelled. Out of 49 patients whose non-emergency appointment was cancelled, all 49 patients underwent transcatheter heart valve replacement or repair later on. Of note, the NYHA class (baseline: 2.4 ± 0.7, intervention: 3.1 ± 0.6; *p* = 0.004) and NT-pro BNP levels (baseline: 1447 [701, 2293] pg/ml, intervention: 2347 [1095, 4279] pg/ml; *p* = 0.006) significantly increased during the time of waiting (Table 3).

**Table 3:** Clinical characteristics of deferred heart valve patients at baseline and at the time of intervention

	Baseline	Intervention	*p* Value
	*n* = 49		
NYHA class	2.4 ± 0.7	3.1 ± 0.6	**0.004**
CCS class	0.5 ± 0.9	0.6 ± 1.1	0.458
NT-pro BNP (pg/ml)	1447 [701, 2293]	2347 [1095,4279]	**0.006**
Troponin T (ng/L)	24 [19, 37|	27 [17, 42]	0.959
LVEF	2.2 ± 1.2	2.0 ± 1.1	0.655
Creatinine (µmol/L)	124 ± 63	122 ± 42	0.851

Bold denotes significant *p*- values

Values are shown as mean ± SD or median [IQR]

*NYHA* New York Heart Association, *CCS* Canadian Cardiovascular Society, *LVEF* left-ventricular ejection fraction

## Discussion

In this study, we have analyzed the outcomes of patients whose non-emergency cardiac intervention had been postponed due to the COVID-19 crisis. We found that the deferral of scheduled cardiac interventions was associated with increased emergency cardiovascular hospitalization or death in the first 365 days, suggesting the progression of disease. This hypothesis is substantiated by our finding of significantly more pronounced symptoms and significantly higher levels of cardiac biomarkers in the study group after 12 months of follow-up. Remarkably, subgroup analyses showed that patients suffering from valvular heart disease, rather than patients scheduled for coronary catheterization or an electrophysiological intervention, experience early emergency cardiovascular hospitalization if their non-emergency intervention is deferred.

Due to the rapidly rising numbers of SARS-CoV-2 infected in spring 2020, hospital resources had to be allocated to sustain IMC and ICU capacities. Consequently, cardiologic societies developed strategies to identify patients who are in a condition allowing to safely defer non-emergency procedures [1, 3, 4].

For example, coronary artery disease patients were deemed suitable for initial medical treatment only in the presence of stable angina up to CCS class III [1, 5]. Furthermore, the deferral of patients with severe aortic stenosis scheduled for transcatheter aortic valve implantation was felt appropriate for compensated patients with an aortic valve area above 0.5 cm^2^ and without recent hospitalization [1, 5]. Postponement of percutaneous mitral valve repair procedures was mostly accepted in the absence of recent heart failure hospitalization [1, 5]. As for patients suffering from arrhythmic heart disease, most procedures were considered to be deferrable, except for immediate life-threatening conditions such as recurrent therapy-refractory ventricular tachycardia, battery replacement in the case of end-of-life in pacing dependency, or the extraction of infected devices [1, 6]. However, recommendations vary depending on the publishing cardiac society.

In our study, we observed that deferred cardiac patients, despite being classified as postponable, show progression of symptoms and experience emergency hospitalizations significantly more often [1]. In contrast, patients who underwent their intervention as scheduled showed an improvement of symptoms at the 12-month follow-up. These findings suggest that the current strategy to manage patients with cardiovascular disease during the COVID-19 pandemic needs refinement and, furthermore, underscores the difficulty of the task shouldered by cardiologic societies to develop general recommendations to avoid overburdened healthcare systems amidst the COVID-19 crisis, while preserving medical care for cardiac patients.

A study evaluating the number of hospitalizations for the different types of cardiac interventions during the first ‘wave’ of the COVID-19 pandemic showed declined interventions in all areas. While the authors observed a reduction in the weekly procedure rate of 20% for transcatheter aortic valve implantations and 28% for percutaneous coronary interventions, the number of pacemaker-, ICD- and CRT (cardiac resynchronization therapy)-implantations declined by approximately 45%. Remarkably, ablations for atrial fibrillation even dropped by more than 80% [7]. These observations possibly reflect the recommendations of the cardiac societies which state that patients with arrhythmic heart disease rather than those with severe valvular heart disease are deferrable [1]. However, studies evaluating the effect of prolonged waiting times on the patients’ course of disease, particularly taking into account the different types of heart disease, are sparse.

Since our study population comprised cardiac arrhythmia patients, patients with severe valvular heart disease and patients with ischemic heart disease, we performed subgroup analyses to evaluate which subgroup predominantly suffered from the prolonged waiting time. Intriguingly, we found that particularly patients with severe valvular heart disease experience early emergency cardiovascular hospitalization if their elective appointment is postponed, while we could not observe significant differences for both coronary artery disease and arrhythmic heart disease patients. These findings are in accordance with the literature.

With regard to arrhythmic heart disease patients, in general, non-emergency interventions for supraventricular tachycardia were considered as being deferrable, since their outcome is rather favourable [1, 6, 8]. Even for patients with documented ventricular tachycardia, it has been demonstrated in the BERLIN VT trial that preventive ventricular tachycardia (VT) ablation before ICD implantation had not reduced mortality or hospitalization for arrhythmia [9]. Interestingly, in the EU-CERT-ICD study, it has been shown that primary prophylactic ICD implantation had been associated with a 27% lower mortality rate [10]. Nevertheless, recommendations did not classify primary prophylactic ICD implantation as either ‘urgent’ or ‘emergency’ and, thus, approved patients’ deferral during the COVID-19 crisis [1]. However, the aforementioned interventions only comprised the minority of arrhythmic heart disease patients included in our study. Most patients were scheduled for catheter ablation for atrial fibrillation and were categorized as ‘lower priority’ during the COVID-19 pandemic [1]. This recommendation is, for instance, referable to the intention-to-treat analysis of the CABANA trial where the authors could not detect a difference in mortality rates between patients undergoing catheter ablation and conservatively treated patients. In contrast, regarding the combined endpoint mortality or cardiovascular hospitalization, they observed a higher event rate in the drug-therapy-only group [11]. In our study, we could only detect a trend towards higher hospitalization rates in patients whose rhythmological intervention had been cancelled. This might be related to the heterogeneity of the cardiac arrhythmia subgroup, their number and the limited follow-up period of 12 months. However, we observed that symptoms of dyspnoea, as measured by NYHA class, significantly increased in the study group and decreased in the control group, underscoring the beneficial effect of a timely rhythmological intervention. Nevertheless, the rather benign outcome suggests that it is possible and reasonable to reschedule non-emergency appointments of patients with arrhythmic heart disease during a pandemic depending on the trend of infection numbers.

Studies evaluating the outcome of coronary artery disease patients with stable angina showed that the outcome of stable CAD patients on a wait list or who are managed conservatively is rather benign [12, 13]. For example, the ISCHEMIA trial demonstrated that an additional invasive intervention is not superior to medical therapy alone regarding both time-to-death and time-to-myocardial infarction [13]. Therefore, stable CAD patients were considered as deferrable during the COVID-19 pandemic [1, 3, 4]. Although we found that the symptoms of patients who underwent coronary catheterization as scheduled improved, and troponin T levels declined significantly compared to patients whose intervention had been postponed, we could not detect a difference in the primary outcome. Consequently, our results suggest that coronary catheterization procedures for patients with stable CAD can be postponed if necessary, however, at the cost of persistent angina pectoris symptoms.

As for patients with severe valvular heart disease, there have been reports that a longer waiting time leads to higher morbidity and mortality. Previous studies have shown that waiting times of 30–80 days are associated with mortality of 2–4.9% in patients scheduled for TAVI [12, 14, 15]. Additionally, after a wait time of almost three months, about 12% of TAVI patients experienced heart failure hospitalization [15]. In patients pending on percutaneous mitral valve repair, studies reported mortality rates of 8% after one and a half months and ≈10% after 180 days [12, 16]. Furthermore, ≈50% of patients were hospitalized for heart failure after 180 days [16]. Here, we demonstrate that patients with severe valvular heart disease are prone to an event of early emergency cardiovascular hospitalization if their non-emergency intervention is deferred. Since the 30-day and 60-day Kaplan–Meier event rates were 34.7% and 53.7%, respectively, our results suggest that especially the first few months following the deferral display the most critical phase in this population. Additionally, the significant increase in symptoms and NT-pro BNP levels during the wait time insinuates relevant disease progression. Consequently, our results substantiate the hypothesis that valvular heart disease patients are especially susceptible to adverse events and the progression of disease if heart valve repair or replacement is delayed, and, thus, might not be suitable for deferral during the ongoing pandemic.

## Limitations

As this is a retrospective observational study on the outcomes of patients with deferred cardiac interventions during the COVID-19 pandemic, it inherently has limitations. Because this is a study from a single center, only a limited number of patients could be included. Additionally, due to the explorative character of this study, our results have to be interpreted as hypothesis generating. Moreover, since recommendations vary by the publishing cardiac society, our approach to identifying patients with deferrable cardiac interventions might be a subject for debate. However, in accordance with the current recommendations of the European Society of Cardiology, only patients who were categorized as ‘lower priority’ or ‘elective’ were chosen to contribute to our study [1]. Moreover, patients with different types of heart diseases were included, resulting in a heterogenous group. Nevertheless, to give an unbiased insight on the effects of deferring cardiac patients during the COVID-19 crisis, all postponed cardiac interventions were included. Furthermore, we observed that patients in the study group had higher NT-pro BNP and troponin T serum levels at baseline. This might display a bias, since patients tended to avoid hospitalization during the pandemic due to the fear of getting infected with the virus. However, these differences did not reach significance.

## Conclusion

To our knowledge, this is the first study comparing the outcomes of patients suffering from coronary artery disease, valvular heart disease, and arrhythmic heart disease, whose non-emergency interventions were postponed during the COVID-19 pandemic. In consideration of our results, we agree with the current recommendations that most patients with stable coronary artery disease and arrhythmic heart disease are deferrable during the ongoing COVID-19 crisis and potential future pandemics. However, we militate against postponing patients suffering from severe valvular heart disease to avoid potential ‘collateral damage’ during the ongoing pandemic. In our opinion, the current recommendations should be revised, since our results at least question the deferability of patients with severe valvular heart disease.

## Supplementary Information

Below is the link to the electronic supplementary material.

Supplementary file1 Supplementary Figure 1 Patients’ NYHA class at baseline and at the end of follow-up in the control and the study group. (B) Patients’ CCS class at baseline and at the end of follow-up in the control and the study group. IQR, interquartile range; NYHA, New York Heart Association; CCS, Canadian Cardiovascular Society (PDF 34 KB)

Supplementary file2 Supplementary Figure 2 Patients’ left ventricular systolic function at baseline and at the end of follow-up in the control and the study group (graded as normal (0), mildly impaired (1), moderately impaired (2) or severely impaired (3). IQR, interquartile range (PDF 26 KB)

Supplementary file3 Supplementary Figure 3 Kaplan-Meier estimators of the time to emergency cardiovascular hospitalization or death of patients undergoing (A) a transcatheter aortic valve implantation (TAVI), or (B) percutaneous mitral valve repair (PMVR) (PDF 58 KB)

Supplementary file4 (DOCX 30 KB)

## Data Availability

The data underlying this article will be shared on reasonable request to the corresponding author.
