# Supplementary figures and images for: Deferral of non-emergency cardiac procedures is associated with increased early emergency cardiovascular hospitalizations
Source: Clin Res Cardiol. 2022 May 23;111(10):1121–9. doi: 10.1007/s00392-022-02032-z (PMC9125015; doi:10.1007/s00392-022-02032-z)

**A**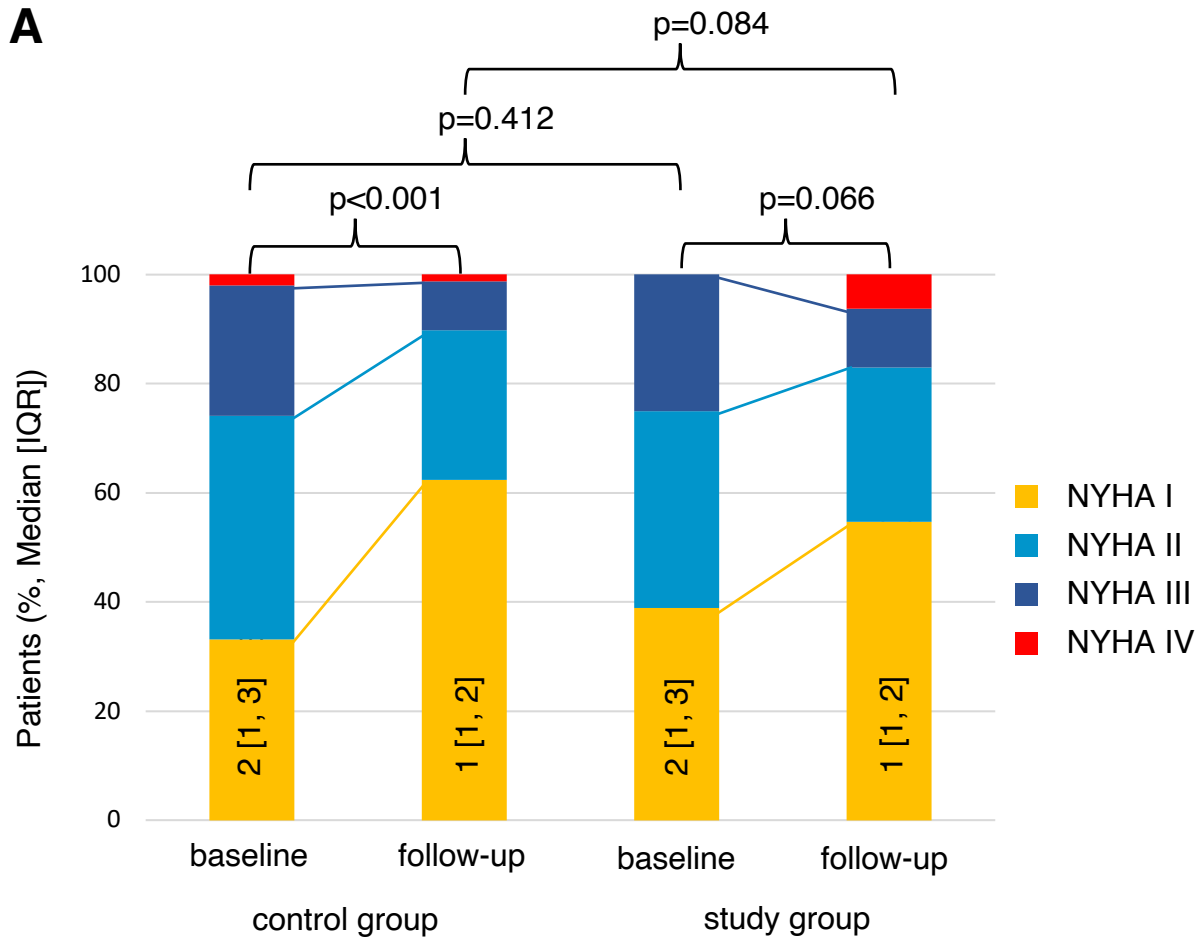**B**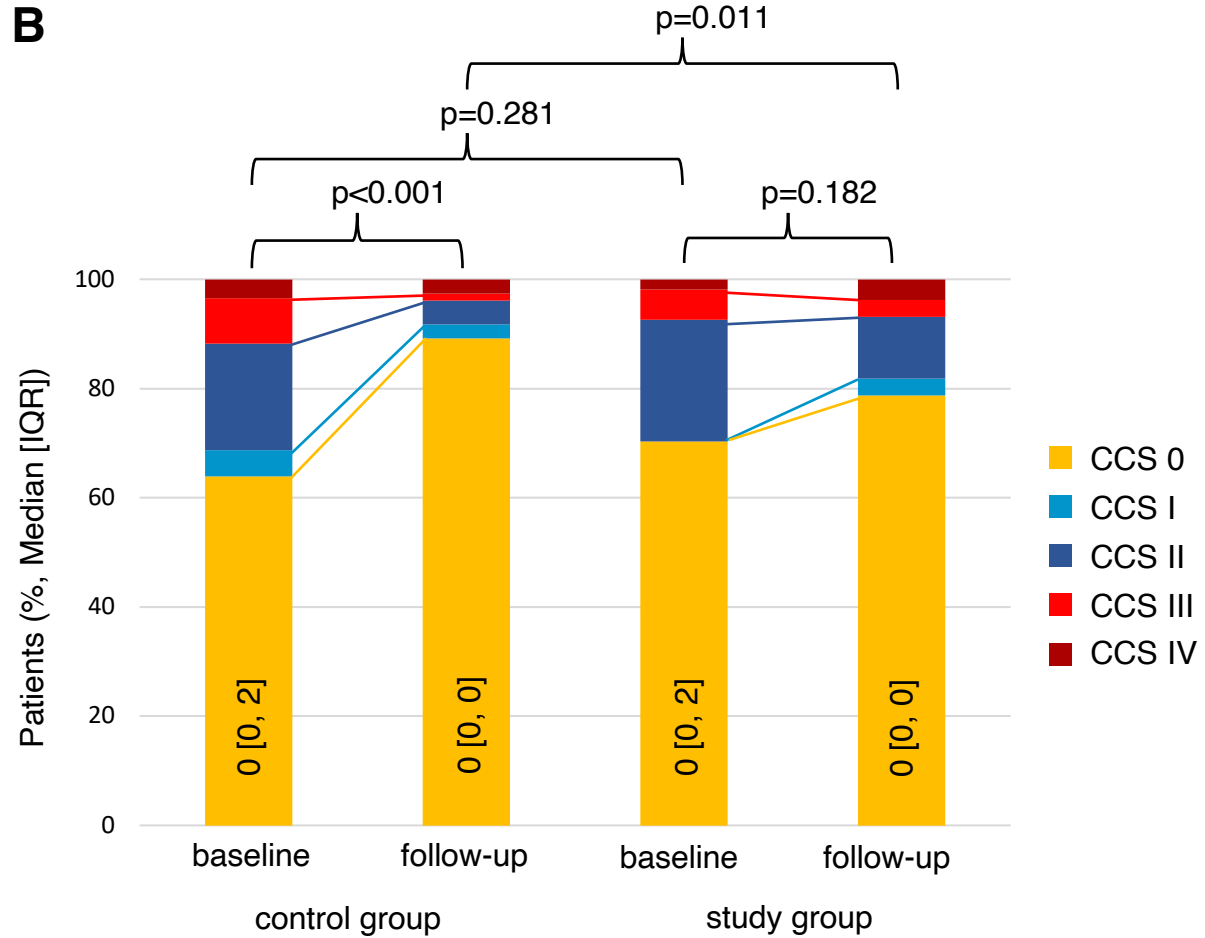

Supplement: Supplementary file 1 — Supplementary file1 Supplementary Figure 1 Patients’ NYHA class at baseline and at the end of follow-up in the control and the study group. (B) Patients’ CCS class at baseline and at the end of follow-up in the control and the study group. IQR, interquartile range; NYHA, New York Heart Association; CCS, Canadian Cardiovascular Society (PDF 34 KB) [file 392_2022_2032_MOESM1_ESM.pdf]

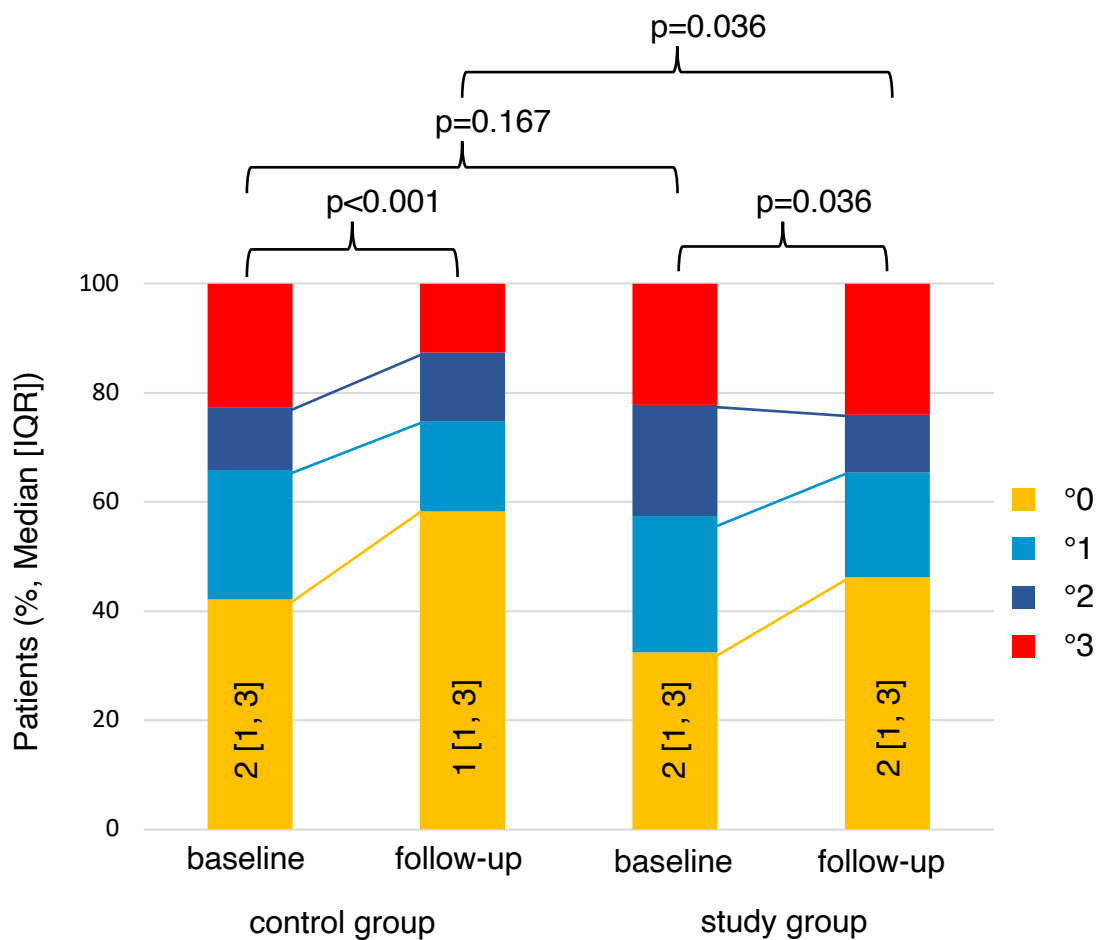

Supplement: Supplementary file 2 — Supplementary file2 Supplementary Figure 2 Patients’ left ventricular systolic function at baseline and at the end of follow-up in the control and the study group (graded as normal (0), mildly impaired (1), moderately impaired (2) or severely impaired (3). IQR, interquartile range (PDF 26 KB) [file 392_2022_2032_MOESM2_ESM.pdf]

**A**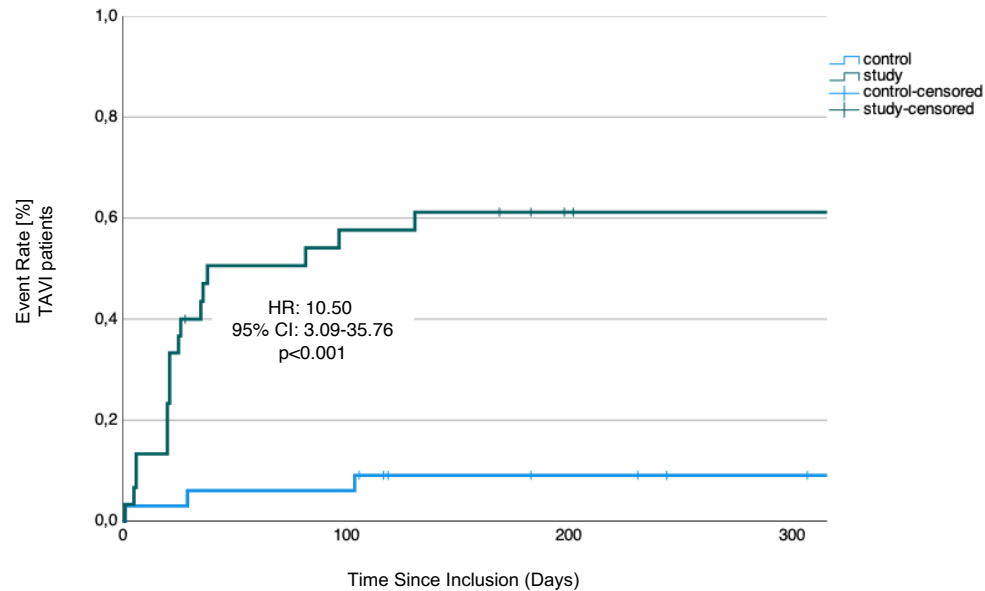

Number at risk:

|               |    |    |    |    |
|---------------|----|----|----|----|
| control group | 33 | 31 | 26 | 24 |
| study group   | 30 | 12 | 8  | 7  |

**B**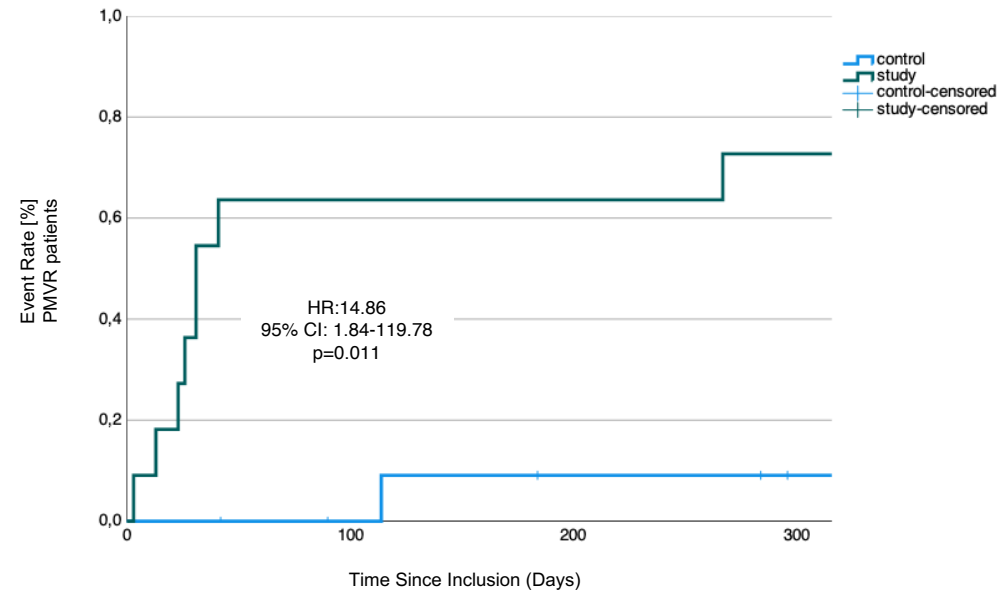

Number at risk:

|               |    |    |   |   |
|---------------|----|----|---|---|
| control group | 13 | 11 | 9 | 7 |
| study group   | 11 | 4  | 4 | 3 |

Supplement: Supplementary file 3 — Supplementary file3 Supplementary Figure 3 Kaplan-Meier estimators of the time to emergency cardiovascular hospitalization or death of patients undergoing (A) a transcatheter aortic valve implantation (TAVI), or (B) percutaneous mitral valve repair (PMVR) (PDF 58 KB) [file 392_2022_2032_MOESM3_ESM.pdf]
