# Supplementary material for: Deferral of non-emergency cardiac procedures is associated with increased early emergency cardiovascular hospitalizations
Source: Clin Res Cardiol. 2022 May 23;111(10):1121–9. doi: 10.1007/s00392-022-02032-z (PMC9125015; doi:10.1007/s00392-022-02032-z)
Supplement: Supplementary file 4 — Supplementary file4 (DOCX 30 KB) [file 392_2022_2032_MOESM4_ESM.docx]

| Supplementary Table 1. Patients’ type of scheduled intervention and type of heart disease | | | | |
| --- | --- | --- | --- | --- |
|  | Control group | Study group | p value | |
|  | n=209 | n=189 |  | |
| Transcatheter heart valve intervention  Aortic valve stenosis  Mitral valve insufficiency  Tricuspid valve insufficiency  Mitral valve stenosis  Aortic valve insufficiency | 48  33  13  1  0  1 | 49  30  11  7  1  0 | 0.147 | |
| Rhythmological cardiac intervention  Atrial fibrillation  Atrial flutter  Other atrial tachycardia  AVRT  AVNRT  VES or VT  EP study only  PM or ICD | 72  40  3  3  1  4  2  1  18 | 63  33  7  0  0  6  7  2  8 | 0.070 |  |
| Cardiac catheterization  Residual stenosis  (Suspected) significant CAD | 89  39  50 | 77  43  34 | 0.122 |  |
| AVRT, atrioventricular reentrant tachycardia; AVNRT, atrioventricular nodal reentrant tachycardia; VES, ventricular extrasystole; VT, ventricular tachycardia; EP, electrophysiological; PM, pacemaker; ICD, implantable cardioverter defibrillator; CAD, coronary artery disease | | | | |

| Supplementary Table 2.  Baseline characteristics of patients undergoing heart valve interventions | | | |
| --- | --- | --- | --- |
|  | Control group | Study group | p value |
|  | n=48 | n=49 |  |
| Age (years) | 79±8 | 80±7 | 0.750 |
| Male sex | 23 (48) | 19 (39) | 0.364 |
| Height (cm) | 165±10 | 174±7 | **0.034** |
| Weight (kg) | 79±21 | 83±18 | 0.654 |
| Heart rate (bpm) | 72±14 | 77±16 | 0.310 |
| Blood pressure systolic (mmHg) | 128±22 | 121±15 | 0.452 |
| Blood pressure diastolic (mmHg) | 70±10 | 68±12 | 0.778 |
| Arterial hypertension | 36 (75) | 23 (79) * | 0.665 |
| Dyslipidemia | 24 (50) | 9 (31) * | 0.103 |
| Diabetes mellitus | 12 (25) | 10 (34) * | 0.372 |
| Family history | 6 (13) | 1 (3) * | 0.181 |
| Smoker | 6 (13) | 7 (24) * | 0.187 |
| Obesity | 12 (25) | 6 (21) * | 0.665 |
| History of TIA/stroke | 3 (6) | 3 (10) * | 0.667 |
| COPD | 4 (8) | 3 (10) * | 1.000 |
| OSAS | 2 (4) | 2 (7) * | 0.629 |
| CKD | 10 (21) | 10 (34) * | 0.186 |
| Known CAD | 32 (67) | 20 (69) * | 0.835 |
| Known cardiac arrhythmia | 23 (48) | 18 (62) * | 0.228 |
| Values are shown as mean ± SD or number (%)  bpm, beats per minute; COPD, chronic pulmonary obstructive disease; OSAS, obstructive sleep apnea syndrome; CKD, chronic kidney disease; CAD, coronary artery disease; * characteristics of 29 patients were assessed | | | |

| Supplementary Table 3.  Clinical characteristics of patients undergoing heart valve interventions at baseline and follow-up | | | |
| --- | --- | --- | --- |
|  | Control group | Study group | p value |
|  | n=48 | n=49 |  |
| NYHA class   - Baseline - Follow-up | ***  2.4±0.7  1.5±0.8 | 2.4±0.7  2.1±1.0 | 0.823  **0.002** |
| CCS class   - Baseline - Follow-up | **  0.9±1.0  0.2±0.7 | 0.5±0.9  0.3±0.9 | 0.187  0.607 |
| NT-pro BNP (pg/ml)   - Baseline - Follow-up | 1645 [865, 4185]  795 [ 548, 2178] | *  1447 [701, 2293]  3307 [2079, 5276] | 0.304  **0.018** |
| Troponin T (ng/L)   - Baseline - Follow-up | 26±19  35±32 | 26±11  48±52 | 0.927  0.472 |
| LVEF   - Baseline - Follow-up | *  2.0±1.3  1.8±1.1 | 2.2±1.2  1.9±1.1 | 0.491  0.628 |
| Creatinine (µmol/L)   - Baseline - Follow-up | 103±36  137±46 | 124±63  142±66 | 0.115  0.820 |
| Values are shown as mean ± SD or median [IQR]  NYHA, New York Heart Association; CCS, Canadian Cardiovascular Society; LVEF, left ventricular ejection fraction; * *p* < .05 versus baseline; ** *p* < .01 versus baseline; *** *p* < .001 versus baseline. | | | |

| Supplementary Table 4.  Baseline characteristics of patients undergoing cardiac catheterization. | | | |
| --- | --- | --- | --- |
|  | Control group | Study group | p value |
|  | n=89 | n=77 |  |
| Age (years) | 69±12 | 71±11 | 0.481 |
| Male sex | 69 (78) | 55 (71) | 0.367 |
| Height (cm) | 171±9 | 172±10 | 0.629 |
| Weight (kg) | 83±16 | 88±19 | 0.152 |
| Heart rate (bpm) | 67±13 | 72±16 | 0.085 |
| Blood pressure systolic (mmHg) | 128±25 | 132±22 | 0.700 |
| Blood pressure diastolic (mmHg) | 83±13 | 76±15 | 0.209 |
| Arterial hypertension | 72 (81) | 59 (80) * | 0.852 |
| Dyslipidemia | 75 (84) | 54 (73) * | 0.077 |
| Diabetes mellitus | 16 (18) | 26 (35) * | **0.013** |
| Family history | 32 (36) | 12 (16) * | 0.005 |
| Smoker | 41 (46) | 35 (47) * | 0.875 |
| Obesity | 21 (24) | 15 (20) * | 0.610 |
| History of TIA/stroke | 7 (8) | 8 (11) * | 0.517 |
| COPD | 5 (6) | 6 (8) * | 0.528 |
| OSAS | 4 (4) | 8 (11) * | 0.124 |
| CKD | 16 (18) | 11 (15) * | 0.595 |
| Known CAD | 66 (74) | 69 (93) * | **0.001** |
| Known cardiac arrhythmia | 32 (36) | 29 (39) * | 0.671 |
| Values are shown as mean ± SD or number (%)  bpm, beats per minute; COPD, chronic pulmonary obstructive disease; OSAS, obstructive sleep apnea syndrome; CKD, chronic kidney disease; CAD, coronary artery disease; * characteristics of 74 patients were assessed, ** characteristics of 51 patients were assessed | | | |

| Supplementary Table 5.  Clinical characteristics of patients undergoing cardiac catheterization at baseline and follow-up | | | |
| --- | --- | --- | --- |
|  | Control group | Study group | p value |
|  | n=89 | n=77 |  |
| NYHA class   - Baseline - Follow-up | **  1.8±0.8  1.5±0.7 | *  2.1±0.8  1.6±0.9 | 0.067  0.751 |
| CCS class   - Baseline - Follow-up | **  1.1±1.3  0.4±1.0 | 1.0±1.2  0.8±1.2 | 0.739  **0.039** |
| NT-pro BNP (pg/ml)   - Baseline - Follow-up | 315 [127, 835]  305 [154, 704] | 791 [226, 1644]  597 [194, 1750] | 0.017  0.141 |
| Troponin T (ng/L)   - Baseline - Follow-up | *  15 [9, 27]  14 [7, 23] | 23 [11, 44]  24 [10, 40] | 0.092  **0.004** |
| LVEF   - Baseline - Follow-up | ***  2.1±1.1  1.8±1.0 | *  2.5±1.1  2.1±1.2 | 0.066  0.112 |
| Creatinine (µmol/L)   - Baseline - Follow-up | 118±137  123±163 | 98±31  102±37 | 0.385  0.349 |
| Values are shown as mean ± SD or median [IQR]  NYHA, New York Heart Association; CCS, Canadian Cardiovascular Society; LVEF, left ventricular ejection fraction; * *p* < .05 versus baseline; ** *p* < .01 versus baseline; *** *p* < .001 versus baseline. | | | |

| Supplementary Table 6.  Baseline characteristics of patients undergoing electrophysiological cardiac interventions. | | | |
| --- | --- | --- | --- |
|  | Control group | Study group | p value |
|  | n=72 | n=63 |  |
| Age (years) | 66±15 | 67±12 | 0.602 |
| Male sex | 47 (65) | 39 (62) | 0.684 |
| Height (cm) | 173±10 | 173±7 | 0.974 |
| Weight (kg) | 86±19 | 84±20 | 0.747 |
| Heart rate (bpm) | 74±20 | 80±17 | 0.126 |
| Blood pressure systolic (mmHg) | 140±3 | 131±19 | 0.323 |
| Blood pressure diastolic (mmHg) | 85±9 | 79±14 | 0.354 |
| Arterial hypertension | 57 (79) | 39 (75) * | 0.584 |
| Dyslipidemia | 49 (68) | 34 (67) ** | 0.871 |
| Diabetes mellitus | 20 (28) | 10 (20) ** | 0.299 |
| Family history | 17 (24) | 10 (20) ** | 0.597 |
| Smoker | 28 (39) | 12 (24) ** | 0.073 |
| Obesity | 19 (26) | 13 (25) ** | 0.911 |
| History of TIA/stroke | 8 (11) | 6 (12) ** | 0.910 |
| COPD | 2 (3) | 4 (8) ** | 0.231 |
| OSAS | 5 (7) | 1 (2) * | 0.399 |
| CKD | 10 (14) | 8 (15) * | 0.816 |
| Known CAD | 40 (56) | 27 (52) * | 0.689 |
| Known cardiac arrhythmia | 64 (89) | 50 (96) * | 0.143 |
| Values are shown as mean ± SD as number (%)  bpm, beats per minute; COPD, chronic pulmonary obstructive disease; OSAS, obstructive sleep apnea syndrome; CKD, chronic kidney disease; CAD, coronary artery disease; * characteristics of 52 patients were assessed, ** characteristics of 51 patients were assessed | | | |

| Supplementary Table 7.  Clinical characteristics of patients undergoing electrophysiological cardiac interventions at baseline and follow-up | | | |
| --- | --- | --- | --- |
|  | Control group | Study group | p value |
|  | n=72 | n=63 |  |
| NYHA class   - Baseline - Follow-up | *  1.8±0.8  1.4±0.7 | *  1.4±0.6  1.5±0.7 | **0.003**  0.647 |
| CCS class   - Baseline - Follow-up | 0.5±1.1  0.1±0.7 | 0.4±1.0  0.2±0.7 | 0.634  0.145 |
| NT-pro BNP (pg/ml)   - Baseline - Follow-up | 1756±3478  2192±6756 | 1962±3221  3815±8019 | 0.791  0.401 |
| Troponin T (ng/L)   - Baseline - Follow-up | 24±44  43±114 | 22±19  85±276 | 0.829  0.480 |
| LVEF   - Baseline - Follow-up | 2.3±1.3  1.8±1.2 | 2.2±1.2  2.4±1.4 | 0.835  0.124 |
| Creatinine (µmol/L)   - Baseline - Follow-up | 99±38  107±40 | 101±68  103±57 | 0.830  0.791 |
| Values are shown as mean ± SD or median [IQR]  NYHA, New York Heart Association; CCS, Canadian Cardiovascular Society; LVEF, left ventricular ejection fraction; * *p* < .05 versus baseline; ** *p* < .01 versus baseline; *** *p* < .001 versus baseline. | | | |
